# Family Health in Adult Education: A Missing Piece of the Health Literacy Puzzle

**DOI:** 10.3928/24748307-20190624-03

**Published:** 2019-10-03

**Authors:** Sara Champlin, Diana Stewart Hoover, Michael Mackert

## Abstract

Previous research and interventions define health literacy as an individual-level concept. Although it is necessary to design programs aimed at individual people, not all health decisions are made by patients themselves, and calls have been made to expand health literacy work beyond the individual. This brief report stems from a larger study in which personnel working for adult literacy coalitions identified family health as a priority topic for health-focused lessons, yet often felt ill-equipped to teach students in this area. This brief report examines adult educators' perspectives on the types of content needed for a family health module designed for the adult education curriculum. Personnel from adult literacy coalitions offered qualitative insights on their desires for health literacy content in the context of family care. Adult literacy coalition educators and staff can provide important insights regarding the health literacy needs of adults in vulnerable populations. Three key themes emerged: American Family Health, Nutritious Eating, and Identify and Act. Rather than using a personal approach, a program that frames health literacy as family health and offers a holistic view on caring for others may serve to provide important context for health decisions and communication for adults at literacy centers. **[*HLRP: Health Literacy Research and Practice*. 2019;3(Suppl.):S75–S78.]**

Health literacy is an individual's ability to find, understand, use, and communicate about health information (Berkman, Davis, & McCormack, 2010). Much research and intervention work focuses on individual-level health decisions and skills. For example, systematic reviews of health literacy outcomes almost exclusively emphasize personal health errors, such as taking medications incorrectly (Berkman, Sheridan, Donahue, Halpern, & Crotty, 2011). Although it is necessary to design individual-level interventions, not all health decisions are made by patients themselves. There is a growing focus on how health literacy is “distributed,” as patients gain health-related support from their social networks and rely on others to help interpret and manage health information (Edwards, Wood, Davies, & Edwards, 2015) Adults may work with family members, friends, neighbors, community leaders, and other informal health information providers (Champlin, Mackert, Glowacki, & Donovan, 2017; McCormack, Thomas, Lewis, & Rudd, 2017). Thus, framing interventions focused on individual care may miss many other situations in which health literacy skills are needed. Calls have been made to expand health literacy work beyond the individual person (McCormack et al., 2017).

In the context of a growing commitment to shared decision-making and active, family-centered care, health literacy efforts may be more effective when positioned in relationship to family health rather than to personal care. Emerging work in the area of health literacy notes that, “Policy makers, government agencies, and community organizations seeking to improve health outcomes in vulnerable populations are paying increased attention to family engagement and health literacy as key elements of a population health approach” (Sivanand, Herman, Teutsch, & Teutsch, 2017, p. 1). The authors argue that cultivating a society involved with health begins with families. By embodying the role a “health advocate” or “partner,” adults can become engaged and committed to the health of their families (Sivanand et al., 2017).

In 2016, the U.S. Department of Health and Human Services (HHS) and the Department of Education issued a joint statement emphasizing family engagement as a key contributor to child health. This statement articulated the important role that early childhood education (including grades kindergarten through 12) and programs such as Head Start, play in building lifelong health literacy skills (U.S. Department of Health and Human Services, 2016). Health literacy initiatives implemented in childhood education are clear choices for skill development; however, it is essential to consider other options for increasing family-level involvement, especially among vulnerable populations.

In an effort to facilitate health literacy and family health engagement within at-risk populations, it is critical to explore the needs of parents who struggle with making decisions and communicating about health topics. Students at adult education or literacy centers are “hard to reach” (McCaffery et al., 2016) and therefore often do not receive existing health literacy interventions. However, these centers, wherein adults develop numeracy and language capacities, offer an important contribution currently missing from the larger HHS initiatives.

Educators and staff at literacy coalitions serve as on-the-ground stakeholders who have a deep understanding of the needs of this audience. With the exception of the contributions of Rudd (2002), health literacy-focused research in the context of adult education centers remains limited (Black, Balatti, & Falk, 2013), despite existing studies that show promising increases in abilities (McCaffery et al., 2016). An important cornerstone for developing programs is to determine what has the greatest “relevance and interest to adult learners” (Muscat et al., 2016, p. 2). At literacy coalitions, educators and staff work in small classes with students, get to know the students well, and in many situations have witnessed the students' struggles with family matters and health topics. As such, these stakeholders can offer important insight on what their students need, value, and desire for family health programs. Although children increasingly receive health content through basic education, initiatives that focus on adults also will contribute to meeting HHS goals.

This brief report stems from a larger study in which personnel working in adult literacy coalitions identified family health as a priority topic that should be included in health-focused lessons, yet they often felt ill-equipped to teach students in this area (Champlin, Hoover, & Mackert, 2018). This article explores adult educators' perspectives on the types of content needed for a family health module designed for the adult education curriculum. This research has several implications, including the identification of family health topics seen as important to adult students, many of whom struggle with health literacy but often feel overlooked in existing programs.

## Methods

### Procedure and Participants

Personnel (educators and staff) at three adult literacy coalitions in Texas created an online survey that solicited views about health content in adult education (**Table 1**). The survey included a notice of consent form, which ensured participants that their responses would be anonymous and that the data would not be used to place or rate their commitment or performance with the coalition. All procedures were approved by the relevant institutional review boards.

### Measures and Analysis

As part of a larger study, participants were asked:

Generally, could you describe what you think students at the literacy center should know when it comes to family health? This can include having healthy relationships, healthy family habits, taking care of children and parents/grandparents, and other topics related to the health of families.”

Participants provided responses and demographic information.

Qualitative responses were printed and explored holistically by the lead author, then reevaluated numerous times using iterative note-taking descriptions, word circling, memoing, and data clustering, as outlined by Hesse-Biber (2017). Inductive thematic codes were developed, beginning with descriptive codes (“tagging” the data), transitioning into categorical codes (grouping descriptive codes), and finally developing analytical codes that gave meaning to the overall phenomenon (Hesse-Biber, 2017). All three researchers were health communication scholars who were trained in qualitative analysis. The researchers discussed the thematic findings and were in agreement that the themes reflected the data.

## Results

Personnel described a number of ideas for family health content in their courses. Nearly all of the points mentioned involved immediate physical health concerns, such as types of care and treatment of illnesses; three themes were identified: American Family Health, Nutritious Eating, and Identify and Act (**Table 2**).

### American Family Health

This theme is about the importance of discussing what family health looks like in the United States. Respondents suggested many adult students balance the culture of their home countries, including speaking in their native language at home and having different expectations of what it means to raise a child, with the expectations and norms presented in American culture. Personnel also believed that cultural norms, including language, might limit how students obtain health information for their family.

### Nutritious Eating

Another area that personnel noted as being important for fostering health literacy in the context of family health was an emphasis on nutrition and healthy eating. From some of the responses, it became clear that incorporating cooking classes in addition to health and nutrition information may be especially helpful.

### Identify and Act

Another critical component for family-focused health content at adult education centers is for adults to become well-versed in identifying ailments, as well as feeling confident about “how to get the help they need.” Personnel hoped students could determine “what to do in an emergency,” as well as when, where, and why they should seek help for loved ones. These responses showed that determining when to access care, as well as how to act, were critical components for family health.

## Discussion

This brief report explored family-engagement health literacy content for adult education centers. Initiatives to facilitate health literacy skills increasingly focus on children (U.S. Department of Health and Human Services, 2016) but traditionally position health literacy as individual-level abilities, despite calls for bigger-picture initiatives (McCormack, Thomas, Lewis, & Rudd, 2017,) and a focus on distributed health literacy (Edwards et al., 2015). Personnel at literacy coalitions work with adults at risk for having low health literacy and therefore can contribute to the “cross-cutting” work needed to move these initiatives forward (McCormack et al., 2017, p.8). Their voice provides an essential contribution to the conversation about health literacy and should not be ignored.

Findings show how health literacy content might be structured in the context of family needs. This includes not only information about specific health topics such as nutrition, but also social and cultural context for health in the United States, which may help facilitate immediate health care needs. Personnel emphasized that connecting students to resources, especially those in their native language and outlining the health care system, might increase students' confidence and contextual knowledge. A module implemented in an adult education context could offer tangible skills and resources in the three themes identified here as well as provide structure for family-based conversations on each topic, thereby addressing the different facets of health literacy and the known role of distributed health literacy in which family and others might share and collaborate on health decisions (Edwards et al., 2015).

Although this study is limited in sample size, it provides insights from three literacy coalitions throughout Texas, thus enhancing the generalizability of the findings. Results provide a bigger picture regarding health literacy needs in adult education by outlining the perspectives of important stakeholders in vulnerable communities. Rather than focusing on content for individual use, personnel offered thoughts on a program that frames health literacy as “family health” and that offers a holistic view on caring for others. This may provide important context for health decisions and communication missing from previous health literacy interventions (e.g., Duren-Winfield, Onsomu, Case, Pignone, & Miller, 2015).

## Figures and Tables

**Table 1:** Participant Demographics (*N* = 47)

**Demographic^a^**	**Composition**

Female	64%

White	72%

Average age (years)	51 (*SD*, 17.94)

Length of time at coalition (months)	29.50 (*SD*, 28.33)

Role at coalition	
Volunteer	62%
Staff	17%
Other^b^	17%

Note.

a When participants indicated ambiguous values such as ranges (e.g., “3–4 years”), an average (e.g., 42 months or 3.5 years) was used.

b Includes AmeriCorps member, community partner, intern, and substitute.

**Table 2:** Participant Quotes

**Theme**	**Quote**
American family health	“Many adult students in literacy programs do not know the American norms—insurance, how to make appointments, the school system (for a child's illness), and helping them feel confident about it is important.” “Parenting is another important topic, especially for immigrant parents who do not speak English well, as expectations in the U.S. are often different than in their home countries, especially around topics like discipline.” “Many of my students might not know much about family health because of the country they come from.”
Nutritious eating	“Leaning how to keep their children healthy is very important, especially learning good nutrition. . .” “Food is a big problem and is not always the healthiest option and not because they cannot afford to eat healthy, but because this is how they were raised to eat and is all they know.” “Taking care of children and eating and cooking strategies. Most of our students are mothers that cook so nutrition is a very important subject.”
Identify and act	“What should they do in an emergency and what to ask the doctor when more information is needed. When they should seek help for each health topic. Why should they seek help rather than just try to treat themselves. Where should they go to be treated for each health topic.” “They should know how to find resources for problems when they need them.” “Try to be aware of signs such as a fever, sweating profusely, anger, distress, and fatigue.” “What to do in case of an emergency.”

## References

[x24748307-20190624-03-bibr1] Berkman N. D. Davis T. C. McCormack L. (2010). Health literacy: What is it? Journal of Health Communication, 15(Suppl. 2), S9–S19. 10.1080/10810730.2010.499985 20845189

[x24748307-20190624-03-bibr2] Berkman N. D. Sheridan S. L. Donahue K. E. Halpern D. J. Crotty K. (2011). Low health literacy and health outcomes: An updated systematic review. Annals of Internal Medicine, 155(2), 97–107. 10.7326/0003-4819-155-2-201107190-00005 21768583

[x24748307-20190624-03-bibr3] Black S. Balatti J. Falk I. (2013). Health literacy and social capital: What role for adult literacy partnerships and pedagogy? Studies in Continuing Education, 35(2), 146–164. 10.1080/0158037X.2012.712038

[x24748307-20190624-03-bibr4] Champlin S. Hoover D. S. Mackert M. (2018). Health literacy in adult education centers: Exploring educator and staff needs. Health Promotion Practice. Advance online publication. 10.1177/1524839918789690 30070148

[x24748307-20190624-03-bibr5] Champlin S. Mackert M. Glowacki E. M. Donovan E. E. (2017). Toward a better understanding of patient health literacy: A focus on the skills patients need to find health information. Qualitative Health Research, 27(8), 1160–1176. 10.1177/1049732316646355 27179023

[x24748307-20190624-03-bibr6] Duren-Winfield V. Onsomu E. O. Case D. L. Pignone M. Miller D. Jr. (2015). Health literacy and computer-assisted instruction: Usability and patient preference. Journal of Health Communication, 20(4), 491–498. 10.1080/10810730.2014.976322 25719814 PMC4462128

[x24748307-20190624-03-bibr7] Edwards M. Wood F. Davies M. Edwards A. (2015). ‘Distributed health literacy’: Longitudinal qualitative analysis of the roles of health literacy mediators and social networks of people living with a long-term health condition. Health Expectations, 18(5), 1180–1193. 10.1111/hex.12093 23773311 PMC5060848

[x24748307-20190624-03-bibr8] Hesse-Biber S. N. (2017). The practice of qualitative research (3rd ed.). Thousand Oaks, CA: Sage.

[x24748307-20190624-03-bibr9] McCaffery K. J. Morony S. Muscat D. M. Smith S. K. Shepherd H. L. Dhillon H. M. Nutbeam D. (2016). Evaluation of an Australian health literacy training program for socially disadvantaged adults attending basic education classes: Study protocol for a cluster randomised controlled trial. BMC Public Health, 16, 454. 10.1186/s12889-016-3034-9 27233237 PMC4884424

[x24748307-20190624-03-bibr10] McCormack L. Thomas V. Lewis M. A. Rudd R. (2017). Improving low health literacy and patient engagement: A social ecological approach. Patient Education & Counseling, 100(1), 8–13. 10.1016/j.pec.2016.07.007 27475265

[x24748307-20190624-03-bibr11] Muscat D. M. Smith S. Dhillon H. M. Morony S. Davis E. L. Luxford K. McCaffery K., (2016). Incorporating health literacy in education for socially disadvantaged adults: An Australian feasibility study. International Journal for Equity in Health, 15(84), 1–10. 10.1186/s12939-016-0373-1 27259476 PMC4893249

[x24748307-20190624-03-bibr12] Rudd R. (2002). A maturing partnership. Retrieved from the National Center for the Study of Adult Learning and Literacy website: http://www.ncsall.net/index.php@id=247.html

[x24748307-20190624-03-bibr13] Sivanand B. Herman A. Teutsch C. Teutsch S. (2017). Building health literacy and family engagement in head start communities: A case study. Retrieved from National Academy of Medicine website: https://nam.edu/wp-content/uploads/2017/04/Building-Health-Literacy-and-Family-Engagement-in-Head-Start-Communities.pdf

[x24748307-20190624-03-bibr14] U.S. Department of Health & Human Services, & U.S. Department of Education. (2016). Policy statement on family engagement: From the early years to the early grades. Retrieved from: https://www2.ed.gov/about/inits/ed/earlylearning/files/policy-statement-on-family-engagement.pdf

